# Changes in the Prevalence of Induced Abortion in the Floating Population in Major Cities of China 2007–2014

**DOI:** 10.3390/ijerph16183305

**Published:** 2019-09-09

**Authors:** Xing Wang, Junqing Wu, Yuyan Li, Ying Zhou, Yiran Li, Rui Zhao, Qi Tong, Mingzhong Luo

**Affiliations:** 1 NHC Key Lab. of Reproduction Regulation (Shanghai Institute of Planned Parenthood Research), Public Health School, Fudan University, Shanghai 200032, China; 2 NHC Key Lab. of Reproduction Regulation (Shanghai Institute of Planned Parenthood Research), Fudan University, Shanghai 200032, China (Y.L.) (Y.Z.) (Y.L.) (R.Z.); 3 Chongqing Population and Family Planning Science and Technology Reseach Institute, Chongqing 400000, China; 4 Shanxi Reproductive Health Technology Service Center, Taiyuan 030012, China

**Keywords:** induced abortion, abortion trends, migrants, contraception

## Abstract

Objective: Our aim was to assess the changes in induced abortion in different migrant groups in China between 2007 and 2014 and the contraceptive methods used prior to induced abortion. Methods: The studies of two population-based cross sections were conducted in urban China, involving 9146 sexually active migrant women. Within the selected sociodemographic subgroups, the changes in the percentage of women who had induced abortions, the proportion of pregnancies ending in induced abortions, the causes of induced abortions, and the methods of contraception were identified. A chi-squared test was used to calculate the differences in induced abortion in the subgroups. Results: Between 2007 and 2014, in the study groups from the major cities of China, the percentage of sexually active migrant women who had induced abortions increased 10.1%, from 21.8% to 24.0%. The proportion of pregnancies ending in induced abortions increased 23.7%, from 21.5% to 26.6%. Both of the aforementioned statistics increased significantly within most of the selected sociodemographic subgroups, especially in the 18–19 and 45–49 age groups. Over 50% of pregnancies were aborted in the cohabiting group, although this figure declined by 12.3% over the course of the seven-year study period. Contraceptive failure was the primary cause of induced abortion, although its contribution to induced abortion declined from 51.3% to 42.4%. The proportion of women not using contraception prior to induced abortion increased from 30.9% to 41.2%. Conclusion: The prevalence of induced abortion is high and continues to increase among sexually active migrant women in China. An increasing trend is forecasted over the next few decades. Special attention should be paid to the younger cohort of migrant women, especially 18–19-year-olds, and unmarried cohabitants, who are increasingly seeking induced abortions.

## 1. Introduction

A few abortions were performed in China before the 1970s. However, in the 1980s, the Chinese government implemented a strict family planning policy to control the excessive population growth. With a few exceptions, such as in areas where the population was sparse, each couple was restricted to having a maximum of one child in order to curtail the population growth rate as quickly as possible. To ensure that the actual number of births met these requirements, some areas adopted extreme and/or compulsory measures. Influenced by the 1994 United Nations Conference on Population and Development and the 1995 World Conference on Women, China subsequently initiated a pilot project on family planning, which was focused on providing quality services based on the premise of informed choice and giving women more options with respect to birth control. In 2010, the prevalence of contraception use in married women in China was 89.20%, ranking the highest in the world [1]. At the end of 2015, the aforementioned strict family planning policy was relaxed, and a two-child policy was implemented. According to the China Health Statistics Yearbook, between 2006 and 2015, the annual number of abortions in China varied from 6,111,375 to 9,851,961, contributing to approximately one-fifth of global abortions [2].

Studies have found that the abortion rate among migrant populations is significantly higher than that among the local residents, accounting for the majority of total induced abortions, especially among young and unmarried women [3,4]. In 2014, the number of the floating population (also known as migrants) in China reached 253 million, more than one-sixth of China’s total population [5]. Moreover, these migrants were predominantly sexually active young people with an average age of 27.6 [5]. This high level of sexual activity combined with a lack of knowledge about sexual health, low income, and difficulties regarding allopatric medical reimbursements has led to an increase in unsafe abortions.

The propensity to have an abortion has been reported to be associated with increased age, lower levels of income, the level of education, migration status, having at least one child and not using effective contraception, and the type of contraceptive use [6,7,8,9]. However, a few studies have analyzed the status of, and changes in, induced abortion among the floating populations across multiple cities. This study focuses on changes in the percentage of women who had induced abortions (the percentage) and the proportion of pregnancies ending in induced abortions (the proportion) among sexually active migrant women in the major cities of China between 2007 and 2014. Furthermore, the causes and public cognition of induced abortion were analyzed, as well as the contraceptive measures used prior to induced abortion.

## 2. Materials and Methods

### 2.1. Patient and Public Involvement

The samples in this study originated from two sampling surveys. According to the results of our preliminary survey, the prevalence of induced abortion among the floating population is approximately 20%. Using alpha = 0.05 (bilateral), beta = 0.10, and design efficiency = 1.5, this study determined that a minimum sample size of 2300 would be needed to conduct a study that was accurate and sufficiently capable of correctly calculating the abortion rate. The inclusion criteria for the survey population were as follows: (1) Female; (2) aged 18–49 years; (3) not registered as a resident in the city where she was working and living; (4) had lived in the current city for at least six months; and (5) was willing to participate in this study.

The first survey was conducted from January to August 2007 in three cities with large floating populations: Beijing, Shanghai, and Chengdu. Considering that the floating population is mainly concentrated in factories, construction sites, and service industries, the random sampling methods were used to select such sites from each city. A cluster sampling method was used at each survey site. From the first survey population, 2333 sexually active women were included as the final subjects of this study. 

The second survey enlarged the scope of the sample and was carried out in Beijing, Shanghai, Chengdu, Hangzhou, and Chongqing in 2014. Just as before, the sample pools of factories, construction sites, and service industries were compiled from each city from which this study randomly selected the survey sites. A cluster sampling method was used at each survey site. A total of 10,634 migrant women volunteered to participate in the study after giving their informed consent, and 6813 sexually active women were selected as the final subjects of this study. 

This study designed two self-administered questionnaires based on the literature review and the reproductive health characteristics of the floating population before the initial surveys. Due to the private and sensitive nature of the questions, each respondent signed an informed consent form and was placed in a separate room to fill out the questionnaire, eliminating peer interference from the process. The two questionnaires took approximately 15–20 min and 30–35 min, respectively, to complete. The investigators were given extensive training to help the respondents complete the questionnaires.

The information gathered by the questionnaire included sociodemographic characteristics, migration history, marriage and childbearing history, sex and health knowledge, informed choice of contraception, and contraception use. With respect to marriage and childbearing history, the respondents were asked about the number of pregnancies, the number of induced abortions, the time of the last abortion, the cause of the last abortion, contraceptive measures used before the last abortion, and their understanding of the health impact of induced abortion. The respondents were divided into different groups by the following characteristics: Age—18–24, 25–29, 30–34, 35–39, 40–44, 45–49; marital status—married, cohabiting but not married, previously married but not cohabiting; educational background—primary school and below, middle school, high school, universities and above; per capita family monthly income—low, lower-middle, middle, upper-middle, high; regional origin—north China, northeast China, east China, southcentral China, southwest China, northwest China. The two main outcome indicators of this study are “the percentage of women who had induced abortions” and “the proportion of pregnancies ending in induced abortion”. The prevalence of induced abortion refers to the former one.

As the second survey was distributed to sexually active women, to make the surveys comparable, the proportions in terms of sexually active females were measured instead of the whole female populations. The two investigations were carried out in several of the same cities among female migrants with the same selected occupations. Their origins were highly consistent. The two additional sites in the second survey are very close to the geographical location of the two sites in the first survey, and their economic level and living habits are also very similar. In order to estimate the status of induced abortion more accurately, this study included all the survey samples. Moreover, the same inclusion and exclusion criteria were used in the study population. Both surveys used structured questionnaires to ask the same questions about induced abortion and other information. The average age of the respondents in the two surveys was similar (33.66 (±8.22) versus 34.88 (±8.44)). The population proportion of each sub-group was also close to each other between the two surveys. All this ensured comparability between the two studies.

### 2.2. Statistical Analysis

The quantitative data from the questionnaires were checked and coded by professional personnel. A database was established with EpiData3.2 software. The frequency distributions were generated based on independent subpopulation statistics for both surveys. The total number of abortions was calculated by the weighted sum of abortions for each woman. A small number of the respondents did not fill in their personal information, such as marital status or family income, and the missing rate of each item was approximately or less than 1%. To avoid deviation caused by unsubstantiated imputation, the rest of the complete data were used to calculate the prevalence of induced abortion in these subgroups. The same method was applied to the total number of induced abortions and pregnancies in each subgroup. 

The percentage of women who had induced abortions = N (women with induced abortion)/N (total women) × 100%

The proportion of pregnancies ending in induced abortion = total induced abortions/total pregnancies × 100%

The changes = (abortion rate 2014 − abortion rate 2007)/abortion rate 2007 × 100%

N (women with induced abortion): The number of women who have experienced abortion through their life time.

A Chi-square test was used to analyze the proportion of women who had induced abortion and the proportion of pregnancies ending in induced abortion with respect to age, marital status, educational background, family income, previous births, investigation site, and the district of origin. SAS 9.4 (v.9.4 SAS Institute, Cary, NC, USA) was used for the statistical analysis. The prevalence maps of induced abortion among sexually active migrant women in China were drawn by the “remap” package in R language. *p* < 0.05 was considered statistically significant. 

### 2.3. Ethical Approval

This study’s protocol was approved by the Research Ethics Committee of the Shanghai Institute of Planned Parenthood Research, WHO (World Health Organization) Collaborating Center on Human Research (Code PJ2014-20) on 29 February 2012. Based on a full explanation of the purpose of the study, informed consent was obtained from all the participants.

## 3. Results

The average age of the participants in the two surveys was 33.66 (±8.22) and 34.88 (±8.44) years, respectively. The percentage of women who had induced abortions increased in most subgroups in the analysis. Overall, 24.0% of sexually active women had an induced abortion in 2014. This is an increase of 10.1% from 21.8% in 2007. The percentage of sexually active women who had another induced abortion increased by 19.4%, from 7.2% in 2007 to 8.6% in 2014. Additionally, 26.6% of the total previous pregnancies ended in induced abortions in 2014, with an increase of 23.7% from 21.5% in 2007 (Table 1). Furthermore, 54.5% and 58.8% of the induced abortions occurred in places of influx in 2007 and 2014, respectively. 

As the study measured the history of induced abortion, the migrants were more likely to experience induced abortion at an older age due to the longer time exposed to an induced abortion. When examined by the age group, the percentage of women who had induced abortions was the highest among women aged 30–34. The percentage was lower in younger or older age groups. The 18–19 and 45–49 age groups showed the greatest increases in percentage. The 20–29 age group declined to different degree. The Chi-square test showed that significant statistical divergence existed between the different age groups with respect to both the percentage and the proportion figures. 

When examined by marital status, the percentage in the cohabiting and previously married groups declined, and the cohabiting group showed the lowest percentage. However, the proportion was much higher in the cohabiting group than in the other two groups. A downward trend was observed, from 60.6% in 2007 to 53.1% in 2014.

The percentage of women who had induced abortions was fairly similar in different education groups in 2007. The percentages were still very close to each other in 2014, except for the universities group, for which the percentage declined dramatically and was significantly lower than the other groups. The proportion of pregnancies ending in induced abortions also reversed the general increasing trend in the universities group.

There was no statistical difference in the percentage of induced abortions among different family income groups in both surveys. The percentage and the proportion both increased the most in the middle family income group. 

Significant statistical differences were found in the previous birth groups with respect to both the percentage and the proportion. These two indicators both declined if the female had not given birth before and increased if the woman had two or more children. Women with no children had the lowest induced abortion rate and the highest abortion–pregnancy proportion, which was similar to the results of the cohabiting group. The percentage and the proportion were higher for women with one girl than for women with one boy.

The origin of the floating population was highly stable in all the survey sites between both surveys (Supplementary Table S1). The percentage and proportion increased the most, nearly doubling, in the Beijing survey site, where the floating population mainly came from northern China. As migrants in Beijing mainly came from northern China, it could be seen that the percentage and proportion among the floating female population from northern China increased the most.

**Figure 1:**
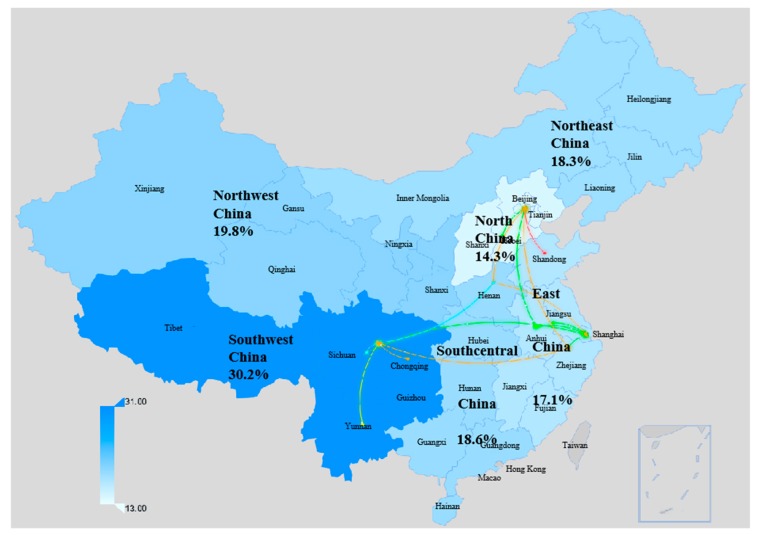
Prevalence of induced abortion among sexually active migrant women by origin of the floating population in China, 2007. The lines represent population migration from the top five sources (provinces) for each survey site.

**Figure 2:**
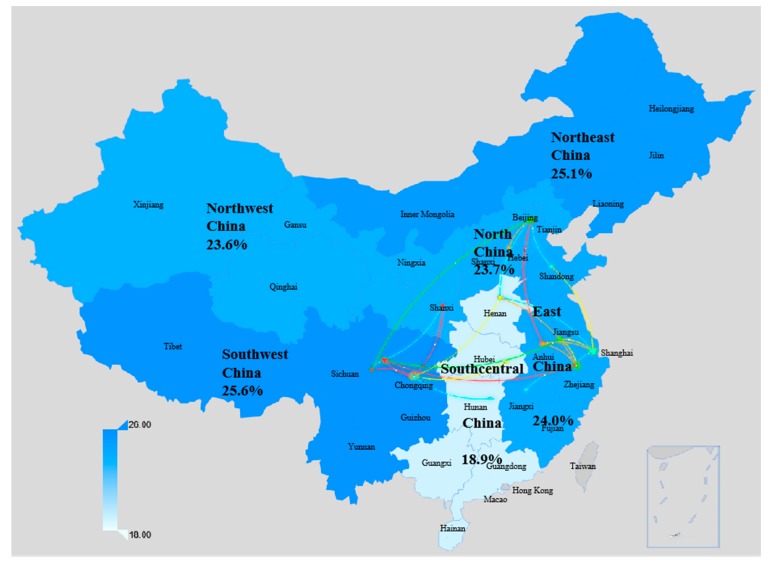
Prevalence of induced abortion among sexually active migrant women by origin of the floating population in China, 2014. The lines represent population migration from the top five sources (provinces) for each survey site.

In 2007, the percentage of women undergoing induced abortion was very similar in each region except for the southwest region, which was significantly higher than that in other regions (Figure 1). In 2014, migrant women from southcentral China had the lowest abortion rate, 18.9%. The rates of the other five regions were fairly close together, ranging from 23.6% to 25.6% (Figure 2). Meanwhile, the abortion–pregnancy ratio of migrant women from southcentral China was the lowest.

The primary cause of induced abortion was contraceptive failure, which declined in recent years, from 51.3% to 42.4%. The second cause was nonuse of contraception, which increased from 30.9% to 41.2%. The principal contraceptive measure used during contraceptive failure was the male condom, accounting for approximately one-half of the total contraceptive failure, followed by intrauterine device (IUD), which accounted for approximately one-third of the total contraceptive failure. Oral contraceptives made up approximately 10% of the total contraceptive failure. A slight increase was observed in the proportional use of male condoms and oral contraceptives and a small decline in the proportional use of IUDs (Table 2).

Most of the respondents firmly opposed induced abortion. However, one-third of the respondents thought induced abortion was acceptable as a remedy for an unwanted pregnancy in 2007, although this proportion declined to 19.0% in 2014. In 2014, the proportion of people who believed that abortion had no impact on health tripled that of 2007 (1.1%), although it accounted for only a small portion of the total population.

## 4. Discussion

Overall, the percentage of migrant women who had induced abortions increased by 10.1% in China from 2007 to 2014. The percentage of women who had another induced abortion increased by 19.4%. This is in contrast to the trends in Estonia, where the percentage of women having repeat abortions has declined dramatically in recent years [10]. A national cross-sectional survey showed that the prevalence of induced abortion was 8.72% in China between 2006 and 2008 [11]. This study revealed a relatively higher prevalence of induced abortion and repeat abortion in the migrant population than in the general population in China. The mean age of the participants in the national study was 27.96 ± 4.10 years (median 27 years), which was much younger than the mean age of the participants (33.66 ± 8.22, 34.88 ± 8.44) in this study. Younger people have shorter exposure to induced abortion, which could partly explain the low rate of induced abortion in the national survey. 

This study found that 21.5% of the total previous pregnancies ended in induced abortion in 2007. In other words, more than one-fifth of the pregnancies ended in induced abortion. However, this proportion increased to 26.6% in 2014. That is to say, more than one-fourth of the pregnancies were aborted, which was consistent with the global average of 25% in 2010–2014 [12].

More than a half of the induced abortions occurred in places of influx, and this proportion was still growing. Reproductive health guidance, such as non-commercial, reliable information on contraceptive methods, should be easily accessible to the floating population in such places of influx [9].

The high percentage of women who had induced abortions reflects the high historical abortion rate [10]. It is expected that women of higher age have a greater chance of having had an abortion, without indication of a higher incidence in that age. The highest percentage was in the 30–34 age group in both surveys, indicating that the abortion rate was increasing for women born during 1960–1980. Moreover, the percentage in the 45–49 age group increased the most, which is consistent with the fact that women born in 1965–1970 began to experience the one-child policy in their reproductive age. Nevertheless, even when the older cohorts of women “age out” of the inclusion criteria, a steady increase in the percentage of induced abortions is likely to occur in the future decades. The same trend has been observed in many countries during the last 30 years [13].

A notable percentage increase in the 18–19 age group indicates that attention should be paid to reproductive health education among the youth. Numerous studies have found that married women have a higher incidence of abortion than unmarried women in China [4,11]. The proportion of marriages within the 20–29 age group in the second survey was higher than in the first survey. One possible reason for the percentage decline in the 20–24 and 25–29 age groups was the increased age for the first marriage in China during 2007–2014 [14].

This study found that people with college or higher education showed a lower propensity to have induced abortions, but their pregnancies were more likely to be aborted. Higher-educated people were more likely to use effective contraception to reduce the risk of unwanted pregnancy, but they also demonstrated less of a desire to have children [15]. 

The proportion of pregnancies ending in induced abortions increased with the increase in family income, which was also caused by less of a desire to have children in the high-income population. A community survey of low-income women in Beijing reported that approximately one-half of low-income urban women aged 18–49 had induced abortions, while 31% had multiple abortions. This study revealed a relatively lower prevalence of induced abortion and repeat abortion in the low-income group [16].

Both the percentage and the proportion were higher in women with one child, especially for women with one girl. Despite the tremendous social development and ideological changes in China in recent years, many families still prefer to have male children. When the first child is a girl, the second child will be probably suffer from sex selection without medical necessity. If the second pregnancy is still a girl, the possibility of induced abortion increases dramatically. The preference for male children has been observed in many other studies [17,18].

Many studies have explored the contraceptive patterns before the termination of pregnancy [19,20], and these variations reflect the overall differences in contraceptive methods in different countries. Condoms generally tend to be the most common method of contraceptive failure before abortion, which is consistent with our findings. Compared with short-term contraceptive methods, the use of long-term reversible contraceptive methods, such as intrauterine devices (IUDs) can reduce the risk of repeat abortions [21]. This was in agreement with our finding that the percentage of IUD users has decreased. With the transformation of China’s family planning policy, young people have generally shown a greater preference for short-term contraceptive methods [22]. IUDs are considered to be the most reliable method of contraception. However, this study revealed a considerable proportion of IUD-caused contraceptive failures compared with Estonia, where IUDs only represented approximately 2% of the total contraceptive use prior to abortion [10]. A previous study in Shanghai reported that 42% of postpartum migrants use IUDs [23], whereas short-term contraceptive methods are the mainstream method of contraception in most of countries [24,25]. The widespread use of IUDs in China has resulted in a high proportion of IUD-induced contraceptive failures. Moreover, the proportion of contraceptive failures has declined in general, indicating that more people are using effective contraceptive measures. The proportion of people not using contraceptives has risen dramatically, suggesting the rise of more casual attitudes toward sex and less concern about the consequences of sexual behavior. In addition, this study shows that the proportion of people who fear poor fetal quality has also increased. This, perhaps, indicates that migrants are attaching more importance to the quality of life of the next generation.

The percentage of women who had induced abortions among those who believed that induced abortion has no impact on health was 61.5% and 40.0% in 2007 and 2014, respectively, which was much higher than in the general population. These women may use induced abortion as a routine contraceptive method, and special attention should be paid to improve their reproductive health awareness. 

## 5. Limitations

This study utilized self-filled questionnaires. Due to the sensitive nature of the topic, some people may have deliberately concealed their histories of pregnancy and abortion, especially unmarried women, even though the authors ensured strict quality control, rigorous training of investigators, and patience and diligence in the investigation process. Moreover, reliance on self-reporting may have led to a memory bias and a tendency to misreport, which may have caused underreporting. As the second survey was conducted mainly among sexually active people, in order to compare the data of the two surveys, the percentage and the proportion among sexually active women were calculated. Thus, there are limitations when comparing these results with the results of other studies. Finally, a small number of missing values were ignored during group computing, resulting in an error of no more than 1%.

## 6. Conclusions

The percentage of women who had induced abortions and the proportion of pregnancies ending in induced abortion all increased during 2007–2014 among 18–49-year-old sexually active female migrants in major cities in China, which was largely caused by the aging out of the older cohort. This increasing trend is expected to last for decades. Special attention should be paid to the younger cohort of migrant women, especially the 18–19 age group; women with one child, especially with one girl; and unmarried cohabitants. This study found that pregnancies in such groups are very likely to end in induced abortion. The proportion of women who think abortion has no impact on health and the proportion of women who do not use contraceptives before induced abortion have risen sharply. Effective measures must be taken to improve migrants’ awareness of reproductive health and the accessibility of contraception.

## Figures and Tables

**Table 1:** The percentage of women who had induced abortions and the proportion of pregnancies ending in induced abortions between 2007 and 2014 among sexually active female migrants in major cities in China.

Characteristic	Number of Women Surveyed		Percentage of Women Who Had Induced Abortions					Proportion of Pregnancies Ending in Induced Abortions				
	2007	2014	2007 (%)	*p*-Value	2014 (%)	*p*-Value	% Change	2007 (%)	*p*-Value	2014 (%)	*p*-value	% Change
Total	2333	6813	21.8		24.0		10.1	21.5		26.6		23.7
Age group				0.002		<0.001			<0.001		<0.001	
18–19	24	139	12.5		20.9		66.9	25.0		38.9		55.6
20–24	250	633	14.0		13.1		−6.3	29.5		23.1		−21.7
25–29	571	1267	23.6		19.1		−19.2	24.8		26.4		6.3
30–34	558	1384	25.1		28.5		13.8	22.2		30.8		38.6
35–39	505	1201	23.4		27.4		17.1	21.5		25.6		19.0
40–44	319	1190	19.7		26.7		35.3	15.7		26.6		69.8
45–49	106	934	15.1		25.1		66.4	10.3		24.2		135.1
Union status				0.052		<0.001			<0.001		<0.001	
Married	2123	6132	22.4		25.3		13.0	20.7		26.1		25.8
Cohabiting, not married	157	481	14.6		11.2		−23.4	60.6		53.1		−12.3
Previously married, not cohabiting	53	200	17.0		14.0		−17.6	18.6		28.4		52.8
Education				0.755		<0.001			<0.001		<0.001	
Primary school and below	318	881	22.3		23.8		6.8	17.4		22.7		30.7
Junior middle schools	1302	3148	21.0		24.3		15.5	20.2		25.2		25.1
Senior high schools/technical schools	604	2216	23.2		25.8		11.4	26.7		30.4		14.2
Universities and above	109	568	21.1		15.5		−26.6	32.4		29.7		−8.1
Per capita family income monthly				0.248		0.267			0.021		<0.001	
Low	283	379	24.4		22.7		−6.9	20.7		24.0		15.8
Lower-middle	506	1621	21.7		22.2		2.2	19.3		22.6		17.1
Middle	754	2535	19.4		24.9		28.3	20.2		27.7		37.1
Upper-middle	451	1334	23.9		24.7		3.0	25.5		27.7		8.8
High	289	872	23.5		25.2		7.2	24.6		30.4		23.5
Previous births				<0.001		<0.001			<0.001		<0.001	
0	332	1508	14.5		9.7		−33.0	69.4		52.6		−24.3
1 boy	821	2180	24.1		27.2		12.8	25.0		29.2		16.9
1 girl	589	1299	27.0		33.1		22.6	26.7		31.9		19.8
≥2	566	1826	17.7		25.5		44.4	11.3		19.1		69.2
Survey sites				<0.001		<0.001			<0.001		<0.001	
Beijing	844	1325	13.7		26.9		96.0	13.8		28.5		105.9
Shanghai	742	1764	20.9		23.6		12.9	20.5		23.5		14.5
Chengdu	747	1045	31.7		17.1		−46.0	28.8		23.3		−19.1
Hangzhou		1775			22.3					23.6		
Chongqing		904			31.9					38.9		
Outflow districts				<0.001		0.004			<0.001		<0.001	
Northeast China	93	183	18.3		25.1		37.5	22.8		34.5		51.0
North China	259	477	14.3		23.7		65.8	13.9		27.1		95.1
East China	737	2594	17.1		24.0		40.5	16.2		23.6		46.0
Northwest China	81	182	19.8		23.6		19.6	22.0		29.2		32.8
Southwest China	825	1889	30.2		25.6		−15.1	28.2		30.7		8.8
Central and Southern China	338	1025	18.6		18.9		1.5	18.6		19.6		5.6

*p*-value: Chi-square test *p*-value.

**Table 2:** The causes of the last induced abortion, contraceptive methods used prior to the last induced abortion, and understanding of induced abortion.

	First Survey (2007)		Second Survey (2014)	
	No.	%	No.	%
Causes of the last induced abortion				
Contraceptive failure	264	51.3	669	42.4
Nonuse of contraception	159	30.9	650	41.2
Fear of poor fetal quality	37	7.2	161	10.2
Other reasons	55	10.7	99	6.3
Contraceptive methods used prior to the last induced abortion				
Male condom	110	48.0	333	51.9
Intrauterine device (IUD)	78	34.1	187	29.1
Oral contraceptive	20	8.7	75	11.7
Other methods (ligation, female contraception, safe period, in vitro ejaculation)	21	9.2	47	7.3
Understanding of induced abortion				
Harmful to health, firmly oppose	1290	56.1	1957	61.0
Harmful to health, but acceptable as a remedy	772	33.6	610	19.0
No impact on health	26	1.1	105	3.3
Don’t know	212	9.2	538	16.7

## Supplementary Materials

The following are available online at https://www.mdpi.com/1660-4601/16/18/3305/s1, Table S1: Top five sources of migrant women.
