# Supplementary material for: Changes in the Prevalence of Induced Abortion in the Floating Population in Major Cities of China 2007–2014
Source: Int J Environ Res Public Health. 2019 Sep 9;16(18):3305. doi: 10.3390/ijerph16183305 (PMC6765927; doi:10.3390/ijerph16183305)
Supplement: Supplementary file 1 [file ijerph-16-03305-s001.pdf]

Table S1. Top five sources of migrant women.

	Survey sites	Sources of migrant women					
		1	2	3	4	5	total
**First survey (2007)**	Beijing<br>844	Hebei<br>228	Henan<br>85	Shandong<br>63	Anhui<br>62	Zhejiang<br>60	498(59.0%)
	Shanghai<br>742	Anhui<br>212	Jiangsu<br>105	Zhejiang<br>73	Sichuan<br>72	Henan<br>58	520(70.0%)
	Chengdu<br>747	Sichuan<br>598	Chongqing<br>44	Yunnan<br>26	Henan<br>25	Zhejiang<br>13	706(94.5%)
**Second survey (2014)**	Beijing<br>1325	Hubei<br>354	Henan<br>155	Shandong<br>143	Sichuan<br>83	Anhui<br>76	811(61.2%)
	Shanghai<br>1764	Anhui<br>488	Jiangsu<br>442	Zhejiang<br>113	Shandong<br>90	Jiangxi<br>86	1219(69.1%)
	Chengdu<br>1045	Sichuan<br>906	Zhejiang<br>24	Jiangsu<br>17	Shanxi<br>12	Chongqing<br>12	971(92.9%)
	Hangzhou<br>1775	Anhui<br>425	Henan<br>359	Jiangxi<br>188	Zhejiang<br>183	Jiangsu<br>100	1255(70.7%)
	Chongqing<br>904	Chongqing<br>471	Sichuan<br>305	Hubei<br>22	Henan<br>19	Shanxi<br>14	831(91.9%)
